# Evaluation of the Implementation of Prenatal Home Visits in Youth Healthcare in the Netherlands: A Mixed Methods Study

**DOI:** 10.1111/jan.16912

**Published:** 2025-03-26

**Authors:** Roos van Tartwijk, Ingrid Staal, Yvonne Vanneste

**Affiliations:** ^1^ Dutch Knowledge Centre for Youth Health, NCJ Utrecht the Netherlands

**Keywords:** advanced practice, antepartum, child protection, community care, health disparities, inequalities in health, nurse roles, nursing home care

## Abstract

**Aim:**

Preventing maternal stress is important for the healthy development of a child. Prenatal home visits were introduced as an integral part of the Dutch preventive youth healthcare for this purpose. This study aims to gain insight into the current state of prenatal home visits.

**Design:**

Mixed methods explanatory sequential design.

**Methods:**

Two questionnaires, one for managers and one for nurses, were distributed to all 38 Dutch youth healthcare organisations. These could be filled in from 29 February to 15 April 2024. Respondents were subsequently invited to participate in an explanatory focus group on 12 June 2024, to help interpret the findings. Questionnaire data were analysed descriptively. Focus group data were analysed qualitatively using open inductive coding. Informed consent was obtained through a privacy statement included with the questionnaire.

**Findings:**

The manager questionnaire had 17 respondents (from 17 organisations), of which 10 participated in the focus group, while the nurse questionnaire had 124 respondents (from 34 organisations), with 8 in the focus group. This study found large differences between youth healthcare organisations regarding collaboration agreements with municipalities, collaboration with referrers, the organisational process and the execution of prenatal home visits in practice. Managers and nurses encounter difficulties with assessing vulnerability in practice and how the intended target group should be reached effectively. While effective collaboration with potential referrers is believed to be essential for achieving accurate and appropriate referrals, the process was experienced as overly complex. No standardised system for documenting data from prenatal home visits was found.

**Conclusion:**

Significant variations exist in organisation and implementation of prenatal home visits across youth healthcare organisations in the Netherlands. The unclear definition of ‘potential vulnerability’ results in an insufficiently defined target group. The lack of standardised data registration hinders the monitoring of the quality, continuity and effectiveness of these visits.

**Implications:**

Variations in organisation and implementation of prenatal home visits may lead to unequal access to care and support for pregnant women and their unborn children across Dutch regions.The lack of a clear definition for ‘potential vulnerability’ results in challenges in identifying and reaching the intended target group, potentially excluding individuals who would benefit most from these services. The absence of a standardised system for data documentation prevents systematic monitoring and evaluation, making it difficult to assess the effectiveness of prenatal home visits and identify areas for improvement. This threatens the long‐term embedding of prenatal home visits in the Netherlands. Further research should also aim to gain insight into the perspective of parents and midwives.

**Reporting Method:**

The paper adheres to the Standards for Reporting Qualitative Research (SRQR) and the Good Reporting of a Mixed Methods Study (GRAMMS) checklists.

**Patient or Public Contribution:**

There was no patient or public contribution to our study.

**Summary**

- Impact
  ○ The problem this study addresses: A lack of knowledge on the implementation of prenatal home visits in Dutch youth healthcare.
  ○ The main findings: There are variations in how the target group is defined and identified, challenges in effectively collaborating with referrers and inconsistencies in managing follow‐up processes. Additionally, the absence of a standardised data documentation system hinders quality assurance and evaluation efforts.
  ○ The research will impact youth healthcare organisations and youth healthcare nurses and ultimately benefit pregnant women and their unborn children.

## 1 Introduction

Maternal stress during pregnancy is widely known to harm a child's development, with both immediate and long‐term effects on their physical and psychological health (Loomans et al. 2013; Van den Bergh et al. 2020). For example, exposed mothers are at a higher risk of delivering newborns prematurely and with lower birth weights. Later in life, prenatal maternal stress has been linked to an increased risk of mental and behavioural issues in the children (Loomans et al. 2013; Van den Bergh et al. 2020). To ensure a healthy development for all children, it is hereby important to prevent maternal stress during pregnancy.

## 2 Background

Dutch youth healthcare (YHC) is a preventive public health system aimed at promoting, protecting and securing the health and physical, social and mental development of children up to the age of 18 years. The YHC is organised nationally and implemented locally via 38 regional organisations, financed and managed by the municipalities. The YHC is offered free of charge to all children and their parents, with an estimated reach of 95% (Vanneste et al. 2022).

At the individual level, personalised basic care such as (anticipatory) information, vaccination and screening is provided through consultations (Vanneste et al. 2022). During these consultations, the care needs are mapped out and preventive support can be offered to children and their families. If deemed necessary, referrals are made to professionals in the medical, social or psychological domain, for example, to receive support related to parenting, lifestyle or developmental delays. The YHC also offers special preventive care to children who grow up in disadvantaged situations, such as children who grow up in poverty or a family with a chronically ill member. Overall, personalised basic care reduces toxic stress, addresses lifestyle and parenting problems and prevents child abuse.

However, the responsibility of the YHC starts before birth. Prenatal home visits were therefore implemented with the goal of preventing health and developmental problems in children by providing early support and interventions to pregnant women in vulnerable situation.

The execution of prenatal home visits has been a common practice for youth healthcare nurses for decades. However, it was never legally established or monitored as it lacked proven effectiveness. As a result, prenatal home visits were scaled down with no insight into where and how they were being executed. This was a concerning development since a variety of positive effects were attributed to prenatal home visits, established in multiple international trials and systematic reviews (Molloy et al. 2021; Dawley and Beam 2005). A recent study showed that an extended home visiting programme in a vulnerable population of pregnant women in Sweden led to more breastfed and vaccinated children and fewer absentees at regular check‐up appointments (Zhe chin et al. 2023). Another study showed that mothers attributed improvements in their parenting skills and emotional well‐being to the support they received during a home visit programme (Zapart et al. 2016).

Prenatal home visits in the Netherlands regained attention in 2018 when the Dutch Ministry of Health launched an action programme called ‘Kansrijke Start’. This action programme aims to ensure that all children have a promising start by targeting the first 1000 days of a child's life, starting at conception. It is strongly believed that events that occur within this window of development greatly influence the opportunities for the remainder of life (Ministry of Health, Welfare and Sport (VWS) 2018; Detmar and Wolff de 2019). In July 2022, a legal amendment was made to the Public Health Law (in Dutch: Wet Publieke Gezondheid), mandating Dutch municipalities to provide prenatal home visits to pregnant women in vulnerable circumstances (Dutch Government 2023). To this end, municipalities receive funding to support and facilitate the implementation and execution of these visits. The amount of funding is determined by the prevalence of preterm and low birthweight deliveries within each region. This also marks the first time that a pregnant woman in a vulnerable situation was defined by law: A pregnant woman with an accumulation of social and psychosocial risk factors that may negatively affect the pregnancy and where protective factors in relation to the woman's self‐sufficiency are inadequate (Wulffraat et al. 2019).

On behalf of the Dutch Ministry of Health, a handbook has been developed by the Dutch Centre of Youth Health (in Dutch: Nederlands Centrum Jeugdgezondheid [NCJ]) to guide the execution of prenatal home visits (Van den Haak and Struijf 2021). This process can be initiated via a referral by a healthcare professional or others involved with the pregnant woman, such as general practitioners, midwives or social workers, who identify sufficient risk factors during a consultation, though pregnant women can also request a prenatal home visit without a referral. The prenatal home visit is conducted by a youth healthcare nurse, consists of one or multiple consultations, and—despite the nomenclature—can be executed in a variety of ways. Besides home visits, it is possible to arrange phone calls, digital contacts or appointments at a youth healthcare location. Through shared decision‐making, the goal is to establish the types of support deemed necessary to remove the pregnant woman from her current vulnerable situation, including follow‐up. After completion of the prenatal home visit, the youth healthcare nurse should report back to the referrer on the completed trajectory and, with the permission of the pregnant woman, its contents.

The change in the position of prenatal home visits, now implemented at a national level, asks for accurate monitoring of the process and its objectives. Studying the implementation process of prenatal home visits is important because their impact is determined by the quality of its application and its reach within the target population. This feedback is essential for ensuring that prenatal home visits continue to improve youth health and well‐being. In June 2023, an initial evaluation of the implementation of prenatal home visits was performed. It revealed that prenatal home visits were widely implemented by youth healthcare organisations (De Craen 2024; Vanneste et al. 2024), and established several areas of improvement. Participants reported that the potential collaboration with referrers should be facilitated and that more guidance from the municipalities was desirable (De Craen 2024; Vanneste et al. 2024). This study reports the second monitor on the implementation of prenatal home visits in the Netherlands.

## 3 The Study

This study aims to examine the current implementation process of prenatal home visits within youth healthcare in the Netherlands as perceived by youth healthcare nurses and managers, with a focus on identifying areas for improvement.

## 4 Methods

### 4.1 Study Design

This study was conducted using a mixed methods explanatory sequential design to allow for a comprehensive evaluation of prenatal home visits (Then et al. 2014). This was done by collecting quantitative (closed questions) via a questionnaire and qualitative data (open questions) through subsequent focus groups. Complementarity, the mixed methods design enabled us to compare the clarification, enrichment, illustration and elaboration of outcomes between the used methods (Greene et al. 1989; Schoonenboom and Johnson 2017).

### 4.2 Theoretical Framework

The Consolidated Framework for Implementation Research (CFIR) (Damschroder et al. 2009) was used to identify factors in five domains that influence implementation: contextual factors (e.g., cooperation with and role of municipalities, availability of data, internal organisation and financial resources), intervention characteristics (e.g., execution of prenatal home visits in practice and perceived added value), professionals involved (e.g., training, skills, collaboration with referrers and motivation of professionals and users) and process characteristics (e.g., quality of training, guidance and monitoring and collaboration with [potential] referrers and the social sector). As with the previous monitor, the CFIR framework guided the development and analysis of the questionnaires. The CFIR framework also guided the presentation of the results to the focus group participants, ensuring that key factors identified in existing research were addressed. To mitigate potential limitations, we included opportunities for respondents to add comments to each answer. Additionally, open‐ended questions and ample space for discussion during the focus groups allowed for the inclusion of perspectives beyond the predefined domains.

### 4.3 Study Setting and Population

The setting of this study is the Dutch preventive youth healthcare, and it was conducted by the NCJ. The study population consists of youth healthcare nurses and managers from all youth healthcare organisations throughout the Netherlands. No limit was set to the number of participants.

### 4.4 Inclusion and Exclusion Criteria

Participating youth healthcare nurses were required to have executed prenatal home visits in practice, and questionnaires that were completed outside of the study period (29 February to 15 April 2024) were excluded.

### 4.5 Recruitment and Data Collection

#### 4.5.1 Questionnaires

In February 2024, two questionnaires were presented to all youth healthcare organisations in the Netherlands (*n* = 38): one for youth healthcare nurses executing prenatal home visits and one for managers. Questionnaires could be completed from 29 February to 15 April 2024 and were spread among the nurses and managers in various ways. Managers received a personalised email asking them to fill in their questionnaire and spread the other questionnaire to three youth healthcare nurses within their organisation. Additionally, the questionnaire for nurses was advertised via multiple NCJ email newsletters and the LinkedIn website. Participation was voluntary.

The questionnaires consisted of open and closed questions covering various subjects regarding the process of prenatal home visits, for example, registration and availability of data (e.g., number of registrations, consultations and follow‐up), internal organisation (e.g., task designation, target group identification, target group coverage, training of nurses, ways for pregnant women to apply and required information for registration), execution (e.g., reasoning behind referrals, registration and follow‐up interventions) and collaboration with (potential) partners (e.g., involvement of municipalities, satisfaction with collaborations). The response options were multiple choice, open or on a visual analog scale (VAS).

#### 4.5.2 Focus Groups

At the closing section of each questionnaire, respondents were invited to participate in a focus group held via videotelephony software Zoom on 12 June 2024, lasting 1.5 h. The goal of the focus groups was to help interpret the results from the questionnaires. Each focus group—one for managers and one for youth healthcare nurses—focused on three subjects to stimulate the conversation, derived by the primary investigators from the questionnaire results. The subjects of the managers' focus group were: internal organisation, collaboration with municipalities and collaboration with birth care organisations (i.e., potential referrers). The focus group with youth healthcare nurses addressed the following subjects: training, collaboration with (potential) partners and the execution of prenatal home visits. Each subject was introduced by sharing the relevant questionnaire results. The focus groups were recorded, for which all participants gave their consent.

### 4.6 Data Analysis

#### 4.6.1 Questionnaires

Data from the questionnaires were analysed descriptively using SPSS, version 29.0.1. The complete research team reviewed the results, after which consensus was reached on which results needed further investigation by means of the focus groups.

#### 4.6.2 Focus Groups

Recordings from both focus groups were transcribed using the programme Amberscript. Participants were anonymised and errors in the written representation of what was said were manually corrected by a researcher. Subsequently, qualitative data analysis was performed using open inductive coding in the programme Atlas.ti (Soratto et al. 2020). Content analysis was performed in two phases: an initial analysis was performed by one researcher, followed by a second round of corroboration and supplementary analyses in consensus with another researcher.

### 4.7 Ethical Considerations

Research in the Netherlands requires approval from a Medical Ethics Review Committee when individuals are subject to specific interventions or behavioural rules, neither of which applied to this study. The privacy of each participant was ethically guaranteed when they agreed to the terms of privacy according to the General Data Protection Regulation (The European Parliament and the Council of the European Union 2016).

### 4.8 Rigour and Reflexivity

Methodological triangulation was used to enhance trustworthiness and broad comprehension of the process of prenatal home visits (Bekhet and Zauszniewski 2012). Results from the questionnaires were reviewed by the complete research team. Besides input on the three proposed themes, it was ensured that enough time was left for participants to address their topics within the focus groups. Additionally, participants were encouraged to speak freely about their experiences. Focus groups were explanatory and were not meant to reach data saturation. Personal biases, experiences, values and assumptions were addressed through open inductive coding and agreement of the analyses by two researchers. In order to improve transparency, the Standards for Reporting Qualitative Research (SRQR) and the Good Reporting of a Mixed Methods Study (GRAMMS) checklists were used for standardised reporting (O'Brien et al. 2014).

## 5 Findings

The questionnaire for managers had 17 respondents, providing information on 17 of the 38 youth healthcare organisations in the Netherlands. Ten managers participated in the focus group. The questionnaire for nurses had 124 respondents from 34 youth healthcare organisations, of which eight nurses participated in the focus group. This section is divided into the four themes covered in the questionnaires: (1) data availability, (2) internal organisation, (3) execution in practice and (4) collaboration. Within each theme, the quantitative results from the questionnaires (closed questions) are presented first, followed by the findings from the open‐ended questions and focus groups.

### 5.1 Data Availability

Not all respondents answered questions regarding general data, such as the expected (*n* = 9) and actual number of prenatal home visits conducted annually (*n* = 15), the number of consultations involved per client (*n* = 10) and the frequency of internal (*n* = 5) and external follow‐up care (*n* = 3). Some of the respondents commented that they only gave rough estimates. Responses in the open text fields revealed that these youth healthcare organisations were unable to supply the requested information due to inconsistent and incomplete registration of prenatal home visits in the digital files.

### 5.2 Internal Organisation

#### 5.2.1 Availability of Employees

In the questionnaire, 13 managers indicated that prenatal home visits are executed by all nurses in their youth healthcare organisations. All 13 managers stated that this choice was based on professionality—the belief that every nurse is capable of conducting a prenatal home visit—and to ensure a seamless transition from pre‐ to postnatal care. In the other 4 organisations, a specialised group of nurses conducts the prenatal home visits. The main reason for this is to ensure that nurses have sufficient experience. One manager expressed concerns that there may be insufficient staffing if the number of prenatal home visits increases in the future:

I also filled out that we have sufficient employees. That is for the amount of home visits we currently conduct. However, we want to expand the amount of home visits we are conducting, but then it gets tight, especially in positioning yourself and networking. (Manager 4)

All managers answered that there are enough employees executing the current demand for prenatal home visits.

#### 5.2.2 Target Group

The majority of managers (*n* = 14) indicated that prenatal home visits are offered exclusively to the intended target group, namely pregnant women in vulnerable situations. Pregnant women are informed about the opportunity of prenatal home visits in various ways by the youth healthcare: through regular consultations (for an older child [*n* = 17] or during the maternal whooping cough vaccination consultation [*n* = 5]), through their website and through flyers (via maternity care contacts [*n* = 13]).

During the focus group discussions, the participating nurses and managers were enthusiastic about the proposal to provide prenatal home visits to all pregnant women. One manager said that everyone is potentially vulnerable, and a nurse mentioned that a prenatal home visit can also serve to encourage parents when things are going well. A nurse stated:

The ideal situation, according to me, would be to offer it to every pregnant woman, so offering it in a low‐threshold way. Then you are no longer just talking about high‐risk pregnancies as the offer stands for everyone. (Nurse 4)

In 3 organisations, prenatal home visits are offered to all pregnant women using the maternal whooping cough vaccination consultation as a catalyst. The focus group participants argued that an advantage of this is the chance that parents are offered the opportunity of a prenatal home visit would not depend on whether they share information with their midwife, nor on the midwife's subjective assessment. One manager mentioned that using the maternal whooping cough vaccination consultation to bring up prenatal home visits has an obvious drawback: this approach misses women who choose not to get the vaccination.

#### 5.2.3 Training of Nurses for Prenatal Home Visits

Based on the manager questionnaire, the training of youth healthcare nurses for prenatal home visits takes place in various ways. In 13 organisations, the prenatal home visit is presented during a work meeting; in 9, training was done via e‐learning; in 10, an internal training was organised; and in 9, a conversation method or an instrument for early detection was introduced. In the focus group, managers brought up that they are still seeking consistent training programmes for new staff. The nurses' questionnaire revealed that they generally feel adequately equipped to conduct prenatal home visits (mean of 7.9 on a VAS scale of 1–10; 1 = not at all, 10 = completely equipped). Besides work experience, what helped most were peer discussions (*n* = 71), completion of the e‐learnings (*n* = 56) and case discussions (*n* = 52). This was confirmed in the focus group. However, the open text fields also revealed that some nurses still have improvement recommendations with regard to their training: this includes access to a clear and broad social map, flyers in other languages for non‐native citizens and education on vulnerabilities commonly seen in practice.

In the managers' focus group discussion, some participants believed that prenatal home visits can be improved by having nurses communicate the benefits of prenatal home visits to both pregnant women and (potential) referrers. Another suggestion was to encourage different nursing teams to exchange experiences with each other:

What we have also been doing is connecting successful nursing teams with nursing teams where things are not going as smoothly, to let them ask each other: how do you offer a prenatal home visit? How do you introduce it? (Manager 2)

Managers emphasised that it is important to continuously remind nurses to communicate about the added value of prenatal home visits, even if they are already doing so.

#### 5.2.4 Prerequisites

Although this topic was not addressed in the questionnaires, managers in the focus group noted that prerequisites necessary for the optimal execution of prenatal home visits were often not met. More specifically, there is a lack of funding for midwives. No time was being allocated to midwives for interviewing pregnant women nor for collaborating with youth healthcare nurses. One manager suggested that national organisations (not further specified) should take responsibility in this:

I really think that those large national organizations must closely look at the funding and collaboration; is that being secured and facilitated? I think there is a real deficit there. In the meanwhile, people from youth healthcare organizations and midwives are working really hard. […] that is currently my biggest expectation and request; I think there is more potential for improvement there. (Manager 6)

The managers questioned whether tasks related to prenatal home visits are included in midwives' job duties.

### 5.3 Execution in Practice

#### 5.3.1 Lack of Referrals

The questionnaires by 15 organisations indicated that a total of 2765 prenatal home visits took place in 2023; 2 organisations estimated 140 prenatal home visits between them. On the other hand, 9 of these organisations had expected to perform a total of 4987 prenatal home visits. In the focus group, several factors were identified by both nurses and managers as contributing to this perceived shortfall in referrals. A nurse suggested that the lower number of referrals could be due to having a different, possibly less vulnerable population in their area. Another reason, mentioned in both focus groups, was that some pregnant women do not feel the need for prenatal home visits. They may respond dismissively or even feel offended by the offer as it can evoke feelings of stigma. One nurse shared that some pregnant women report feeling overwhelmed with information, or view a home visit as intimidating. A nurse added:

Parents may have an attitude such as: why do I need to have a prenatal home visit? What's wrong? They find it difficult to talk about. (Nurse 6)

A third point was the presumed influence of potential referrers on the number of referrals. Both nurses and managers noted that referrers sometimes fail to recognise the added value of prenatal home visits. Nurses shared that midwives often communicate that they see no need to involve an additional person. Moreover, referrals are frequently made only in cases of multiple significant problems or later in pregnancy, possibly because a trusting relationship needs to be established before parents feel comfortable sharing their vulnerabilities. Nurses emphasised that earlier, low‐threshold referrals would also be appreciated.

Additionally, some managers and nurses believe that there may be a negative attitude among midwives towards youth healthcare organisations. Managers suggested that dissatisfaction among midwives regarding previous decisions about the division of roles between youth healthcare professionals and midwives could influence their attitudes. Several nurses also mentioned that midwives might perceive youth healthcare as a rival. A nurse stated:

They shouldn't see us as a rival, but that's all about gaining trust. (Nurse 3)

Some nurses mentioned in the focus group that there are sometimes a lack of trust and unfamiliarity in each other's expertise.

#### 5.3.2 Process of Signing Up

The managers' questionnaire revealed that there are several ways to sign up for a prenatal home visit: by phone (*n* = 15), through the website (*n* = 12), at a youth healthcare location (*n* = 11) or by email (*n* = 8). One manager mentioned in the focus group that she had noticed the process of signing up can sometimes be unclear for referrers. Many nurses in the focus group shared that, in practice, signing up is almost always done by the referrer (with the pregnant woman's consent).

All managers reported that their sign up forms ask for the contact details of the pregnant woman (*n* = 17). Most also ask for the contact information of the referrer (*n* = 14) and a brief explanation for the request (*n* = 9). In the focus group, some nurses expressed a desire for additional information from the sign up process, such as the preferred spoken language.

#### 5.3.3 Prenatal Home Visits and Follow‐Up

The nurses' questionnaire revealed that the most common reasons for referrals were the presence of multiple problems (*n* = 74), physical or psychological health concerns (*n* = 70), financial and housing hardships (*n* = 55) and pregnant women's insecurities about caring for their child (*n* = 44).

According to the nurses' questionnaire, contact with pregnant women was predominantly made through home visits (*n* = 778). Other forms of contact included conversations at a youth healthcare location (*n* = 55) or via teleconferencing (*n* = 28). There was considerable variation across nurses as to whether a single prenatal home visit was sufficient. In the focus group, most nurses reported using various tools, methods or programmes to guide their conversations with pregnant women. One nurse explained that these tools are useful for keeping the conversation broad and open:

I find the use of a conversation methodology helpful. It helps to start a conversation in an open way and to discuss all aspects; to be thorough. (Nurse 2)

Some nurses choose not to use any specific tools to guide the conversation; instead, they rely on their professional experience.

Nurses' questionnaire responses indicated that follow‐up interventions were most frequently initiated in areas related to financing and housing (*n* = 56), health (*n* = 48), parental insecurities regarding child care and upbringing (*n* = 38) or a combination thereof (*n* = 53). When asked whether it was possible to implement the appropriate follow‐up interventions, different comments were made in the focus groups. According to participants, the availability of interventions often depends on the municipality. It is sometimes unclear which interventions are available, and long waiting lists are common in the social sector (such as for debt assistance or family‐focused home care). Some managers in the focus group highlighted that the absence of formal agreements to bridge service gaps can make this process particularly challenging, leading to frustration among nurses.

If no follow‐up intervention is available and there are no agreements for bridging the gap in care, tension arises for nurses between workload on the one hand and sense of responsibility on the other. (Manager 10)

Managers' responses from the questionnaire showed that only 2 out of the 17 organisations have agreements with municipalities about bridging gaps in care if no follow‐up care is available.

#### 5.3.4 Registration and Completion

The questionnaire revealed that 74 nurses document their prenatal home visits in parents' files, while 39 record these in the children's files. In the focus group, some nurses indicated a desire to link the parent's file to the child's file to enhance continuity of care from the prenatal to the postnatal period.

Nurses usually provide a report on the process and content to the referrer following the completion of a prenatal home visit (mean of 7.2 on a VAS scale of 1–10; 1 = never all, 10 = always). However, reports are not shared when no report is written, when the pregnant woman did not consent to the disclosure of information, or when the pregnant woman signed up for the prenatal home visit herself. In focus groups, both managers and nurses emphasised that reporting on the process and content enhances the quality of communication with referrers:

We hear that reporting to the referrer is not always done or not always done well enough. What is said is: “If we midwives don't get reports, it doesn't motivate us to make a referral the next time”. So that's an important key issue to get right; pick it up and provide reports to the referrer. (Manager 5)

The questionnaire showed that nurses believe that prenatal home visits offer several advantages. The greatest advantages are the earlier detection of vulnerabilities with faster deployment of help (*n* = 112), a good way to initiate the first contact with youth healthcare (*n* = 109), continuity from pre‐ to postnatal care (*n* = 91) and strengthened collaboration between maternity care, youth healthcare organisations and the social sector (*n* = 74).

### 5.4 Collaboration

#### 5.4.1 Collaboration With Municipalities

The managers' questionnaire indicated considerable variation in municipal involvement in facilitating prenatal home visits (mean of 6.1 and range of 3–10 on a VAS scale of 1–10; 1 = not involved at all, 10 = very involved). The role that municipalities take on differs tremendously, for example with regard to facilitating local coalition meetings and the availability of follow‐up interventions. The majority of managers (*n* = 15) reported that their organisation has a procurement agreement with an effort‐based commitment, while 2 managers indicated having a procurement agreement with a results‐based obligation. Overall, managers generally reported receiving adequate funding to support the current volume of prenatal home visits. Two relevant comments were: ‘With the current numbers of prenatal home visits, we are well off with the financial resources. As numbers increase we will have to discuss this’ and ‘The home visits lead to additional follow‐up activities for which there is not always budget or time.’

The managers' questionnaire identified several factors that encourage municipalities to actively take up their role regarding the support of prenatal home visits. This includes enhancing knowledge and understanding of the benefits of prevention, facilitating network collaboration through a local coalition, maintaining the continuity of municipal officials and establishing clear and direct communication channels. Most managers indicated that they regularly place prenatal home visits on the agenda in local coalition meetings, which were perceived in the focus group as contributing positively to collaboration. However, some managers remarked that the status and development of prenatal home visits in these local coalitions vary significantly across municipalities:

Some coalitions are currently not functioning due to a variety of reasons. Where one coalition is very progressive and has been functioning and growing for years, the other is just starting up or has been going through a difficult time, causing the prenatal home visits to receive less attention. I see that it just varies a lot. (Manager 10)

Managers' questionnaire identified several potential barriers to collaboration between municipalities and youth healthcare organisations, such as lack of knowledge and awareness about prenatal home visits, high turnover of officials and excessive workloads. One manager explained in the focus group that, at times, municipalities do not adequately fulfil their role:

I do find it difficult. It is a legal duty, but what is my power if a municipality does not take that seriously enough? Or says that there are no more resources to purchase more interventions? Then I don't get much further than saying that is how it is. (Manager 4)

Managers are unsure how to approach municipalities that do not fully meet their responsibilities in facilitating prenatal home visits.

#### 5.4.2 Collaboration with Referring Partners

The answers in both questionnaires showed that satisfaction with the collaboration with referring partners is experienced as very variable (mean of 6.7 and range of 4–9 for managers (*n* = 17) and mean of 6.6 and range of 2–10 for nurses on a VAS scale of 1–10; 1 = very negative, 10 = very positive). This was confirmed in the focus groups, where participants named various factors that they considered crucial for effective collaboration.

First, both managers and nurses emphasised the importance of ensuring that referrers and potential referrers have adequate awareness and understanding of prenatal home visits. According to them, the opportunity for a prenatal home visit is often insufficiently recognised, and there is a lack of understanding regarding what it involves and the benefits it offers. Second, regular contact plays a crucial role in maintaining awareness of prenatal home visits. Several managers and nurses in the focus groups also pointed out that multidisciplinary consultations, such as the VSV (Dutch midwifery partnership) and POP‐poli (outpatient clinic for pregnant women with psychological vulnerabilities), contribute to improved collaboration. A third key factor for successful collaboration is building a network with potential referrers. Both managers and nurses highlighted that building a trusting relationship takes time, and investing in collaboration is a continuous process:

It really is building, that is how I see it. We seek more and more connections on all kinds of levels, but it is really a continuous process. It is not finished tomorrow. (Manager 10)

Zooming in on the various potential referring partners, both managers and nurses indicated that midwives are key referral partners. Participants reported differences in collaboration among obstetric practices; some practices consistently provide referrals while others offer few or none. This lack of referrals may stem from a failure of the practice to fully recognise the value of prenatal home visits. A nurse explained that a limiting factor for effective collaboration is a lack of time:

We don't have many consultations with midwives, but that is really due to a lack of time. I can imagine that if you do that more often, the collaboration will improve. (Nurse 5)

Nurses also mention that they deal with a large number of obstetric practices across a broad geographical area and that this is a limiting factor for effective collaboration.

As for other potential referrers, the nurses expressed that contact with general practitioners is still lacking, with the number of referrals remaining far below their expectations. Regarding collaboration with hospitals, managers in the focus group mentioned that it is often challenging to find the appropriate contact person. Some managers noted that organising prenatal home visits in a larger geographical area can be difficult, as hospitals then have to coordinate with multiple youth healthcare organisations.

#### 5.4.3 Collaboration with the Social Sector

Managers and nurses did not identify the social sector as a potential referrer, but primarily as a sector for follow‐up interventions. Regardless, the current relationship between prenatal home visits and the social sector remains unsatisfactory:

It is unclear what follow‐up interventions are available and there is often a waiting list in the social sector. (Nurse 3)

In the managers' focus group, it was viewed that the participation of the social sector in local coalitions would be beneficial for successful collaborations.

#### 5.4.4 The Role of the NCJ

Although it was not addressed in the questionnaires, the role of the NCJ in the implementation process of prenatal home visits was raised during the managers' focus group. One manager suggested that the NCJ could play a role in fostering connections between national organisations by arranging a joint webinar for all potential referrers, for example. Another manager proposed that the NCJ could help establish a funding model to support the collaboration and involvement of midwives.

##### 5.4.4.1 Case 1

A youth healthcare nurse received a referral for a prenatal home visit from a midwife. The midwife provided the nurse with the expectant mother's phone number, email address and a brief explanation for the referral, stating that the mother appeared to have difficulty understanding her pregnancy. The nurse contacted the expectant mother and arranged a prenatal home visit, but the mother was not at home on the day of the appointment. It appeared she did not know about the scheduled visit. A new appointment was set and, during the first conversation, the nurse learned that the expectant mother—a 30‐year‐old married woman living alone while her husband awaited a residence permit—had a limited understanding of the situation. Further questioning revealed that she had been diagnosed with an intellectual disability. Recognising the vulnerability of the situation, the experienced nurse followed up with multiple home visits. The nurse contacted the midwife, helping her understand that the expectant mother's failure to attend appointments was due to low literacy, not a lack of commitment. The nurse believed the mother was capable of learning but needed clear instructions and structure to care for her child. To support this, the nurse initiated two follow‐up interventions: education about pregnancy and childbirth and practical tools to help the mother organise childcare tasks. Despite initial challenges after childbirth, the mother was able to care for her child at home with daily support provided.

Reflecting on the experience, the youth healthcare nurse emphasised the added value of the prenatal home visit. She noted that obstetric care was improved because appointments could be made in a way that the expectant mother could understand. The visit allowed for the timely deployment of appropriate interventions, giving the mother a real opportunity to care for her child at home.

##### 5.4.4.2 Case 2

A woman, pregnant with her fifth child, was signed up for a prenatal home visit by her midwife through the local youth health organisation's website. The woman had moved to the Netherlands 1.5 years ago, and this would be her first delivery in the Netherlands. During the prenatal home visit, the youth healthcare nurse assessed her situation and identified several vulnerabilities: the pregnancy was complicated due to gestational diabetes and, in addition to caring for her four children, the woman also looked after her husband who had suffered a stroke. The family had limited support, was facing financial difficulties and the mother did not speak Dutch. The nurse identified the practical matters that still needed to be arranged before delivery and took the opportunity to highlight the mother's strengths, empowering her by acknowledging how she was managing the challenges she faced. In collaboration with the midwife, the nurse ensured that all necessary preparations were made before the birth. After the delivery, the same nurse conducted the regular postnatal check‐up to maintain continuity of care from pregnancy to postpartum. The prenatal home visit, which also served as an introduction to youth healthcare, helped build a trusting relationship between the mother and the nurse. This example of trust enhances the quality and effectiveness of youth healthcare. It ensures that mothers feel listened to, understood and supported—key factors for the well‐being and health of both mother and child.

## 6 Discussion

The aim of this study was to gain insight into the current state of prenatal home visits in youth healthcare in the Netherlands. This was done through analyses of questionnaires and focus groups with youth healthcare nurses and managers from youth healthcare organisations throughout the Netherlands. This study found that there are large differences between youth healthcare organisations regarding data registration, internal organisation, execution in practice and collaboration with municipalities and referring partners. These results are discussed under the themes of *Lack of Referrals* and *Quality of Execution*.

### 6.1 Lack of Referrals

This was evident from both the questionnaires and the focus groups that the number of referrals was lower than expected. The main factors that could be of influence on the perceived lack of referrals are discussed here: the concept of potential vulnerability, the target group approach and collaboration with referrers.

#### 6.1.1 The Concept of Vulnerability

The presumption by managers and nurses that referral rates of potentially vulnerable pregnant women are insufficient cannot be substantiated. Firstly, accurate referral numbers remain indeterminable due to inadequate data registration. Secondly, the estimated number of expected referrals is extrapolated from the population of children in the Netherlands who encountered adverse early life conditions due to preterm birth or low birth weight. However, not all instances of vulnerability result in preterm birth or low birth weight, nor are preterm birth and low birth weight exclusive outcomes of vulnerable circumstances. Therefore, the validity of this estimate as an accurate reflection of the number of vulnerable pregnant women is highly questionable. Thirdly, this study highlights that the current definition of a ‘potentially vulnerable pregnant woman’ lacks clarity and practical applicability. Vulnerability is theoretically already a complex concept and, in practice, it extends beyond a simple imbalance of risk and protective factors (Feijen‐de Jong et al. 2021). Both managers and nurses express uncertainty about operationalising the concept of vulnerability in practice. Some suggest that all pregnant women could be considered potentially vulnerable due to the challenges that accompany childbirth. This perspective advocates that youth healthcare organisations should offer prenatal home visits to all pregnant women.

#### 6.1.2 The Target Group Approach

One advantage of offering prenatal home visits to all pregnant women is that it removes their reliance on potential referrers, especially when no trust has (yet) been established. Additionally, this approach could help normalise the concept of prenatal home visits. This is in accordance with the observation that some pregnant women perceive these visits as stigmatising or intimidating, aligning our findings with those from previous research (Rutz et al. 2024). This potential barrier may be lessened by extending the offer to all pregnant women, enabling better access to the intended target group (regardless of who that target group may be).

Some organisations introduce prenatal home visits during maternal whooping cough vaccination appointments as a strategy to reach all pregnant women. However, current maternal whooping cough vaccine coverage in the Netherlands stands at approximately 70% (Immink et al. 2023). Research indicates that individuals with lower literacy levels and socioeconomic positions are more likely to decline vaccination (Anraad 2023). This raises concerns regarding the effectiveness of utilising the maternal whooping cough vaccination appointment as a means to engage the most vulnerable populations in prenatal home visit initiatives.

#### 6.1.3 Collaboration With Referrers

In current practice, effective collaboration with potential referrers is considered essential for increasing referral rates. This process is complex and requires ongoing investments from all involved parties to establish a strong network and promote prenatal home visits. A review by Hope Corbin et al. (2018) underscores that successful intersectoral collaboration for health promotion relies on multiple key elements, including building trust among partners and conducting regular evaluations to foster continuous improvement in partnership effectiveness.

According to managers and nurses, collaborations with hospitals, general practitioners and social service providers as referring partners are currently ineffective. A key challenge is a misalignment between hospital service regions and those of youth healthcare organisations. Furthermore, nurses have to deal with a large number of potential referrers, making collaboration a time‐consuming responsibility that is not accounted for in the funding of prenatal home visits. While the municipalities' involvement in a facilitative role supports collaboration with referrers, the extent to which the municipalities fulfil this role varies considerably. Previous research suggests that municipalities can support this effort by adopting a development‐oriented approach in their management practices (Rutz et al. 2024).

### 6.2 Quality of Execution

There is variation in how nurses prepare for conducting prenatal home visits. Though this is not an issue, it underscores the benefits of offering diverse training methods to suit each nurse's preference. Additionally, managers emphasise the importance of communicating the added value of prenatal home visits to pregnant women and potential referrers; this would ideally be an integral part of the nurses' training. Both managers and nurses agree that work experience is important, which has led some organisations to appoint qualified nurses as ‘prenatal home visit specialists’. Finally, nurses regard case discussions as a vital component of their training. Supporting this, a study by Popil found that learning through real‐life case analysis fosters active learning while enhancing the nurses' critical thinking, problem‐solving and emotional preparation for the real world (Popil 2011).

This study revealed that, despite positive recommendations (Van den Haak and Struijf 2021), no risk assessment instrument is integrated into prenatal home visits. This is despite the fact that the feasibility of such tools in daily practice has been demonstrated through the support of professionals in determining appropriate follow‐up interventions (Van Driessche et al. 2021).

Youth healthcare nurses recognise several benefits of prenatal home visits for pregnant women. The most important perceived benefits are early identification of vulnerabilities, faster deployment of support services, establishing initial contact with youth healthcare, ensuring continuity between pre‐ and post‐natal care, and fostering stronger collaboration between maternity care, youth healthcare organisations and social service sectors.

However, the perceived added value of prenatal home visits reported by nurses remains difficult to verify objectively. Due to the lack of standardised documentation, it is unclear how often prenatal home visits are conducted, which activities are performed during these visits, and what the effectiveness of these visits is in meeting their intended objectives.

### 6.3 Recommendations

#### 6.3.1 Recommendations for Practice

To assess the quality of prenatal home visits and their effectiveness in achieving their goals, we strongly advocate standardised data registration in all youth healthcare organisations in the Netherlands. It is also essential to achieve consensus on who the target group is and the main strategy to reach them. Without a clear and consistent framework, it will be impossible to provide clear guidelines for the implementation of prenatal home visits or to evaluate their effectiveness in achieving their intended goals. Failure to demonstrate the effectiveness of prenatal home visits could ultimately lead to budget cuts.

We also recommend revising the financial structure of prenatal home visits. There is currently no funding allocated to time spent on the training of nurses and collaboration with referring partners. Funding these activities would respectively improve the quality of prenatal home visits and improve the strength and number of partnerships. It is also important that municipalities recognise their crucial role in facilitating the collaboration between referrers.

#### 6.3.2 Recommendations for Further Research

We recommend the continued monitoring of prenatal home visits in the future. For further research, it is essential to establish outcome measures that accurately reflect the effectiveness of prenatal home visits. This is crucial for objectively assessing whether these visits are successful in achieving their primary goal: preventing health and developmental issues in children by providing early support and interventions to pregnant women in vulnerable situations.

We also suggest that further research should incorporate the perspectives of pregnant women and their partners, including those who have received a prenatal home visit as well as those who have not. Such an investigation could offer valuable insights on how to more effectively reach the intended target population. Additionally, we recommend exploring the perspectives of midwives regarding the prenatal home visit process. Given that midwives are identified by managers and nurses as the primary referrers, improving collaboration with them is critical for enhancing referral rates.

### 6.4 Strengths and Limitations

This study has a number of strengths, including the complementarity of qualitative and quantitative data collection through the questionnaires and focus groups. It has allowed us to gain a broader understanding of the perspectives of youth healthcare managers and nurses on the current process of prenatal home visits. This analysis offers a more detailed examination of the prenatal home visit process compared to the initial evaluation from 2023 (Vanneste et al. 2024). This study included more comprehensive questionnaires with in‐depth questions, and data from focus groups were transcribed and systematically coded for thorough analysis.

One of the limitations of this study is that information is not available from all youth healthcare organisations in the Netherlands. The questionnaires were not validated but were developed based on results from the previous monitor. Also, the open text fields enabled any necessary elaboration.

Another limitation is that focus groups were solely explanatory and not meant to attain data saturation. However, the focus group approach was chosen to allow participants to explain the questionnaire results, fostering interaction and diverse insights while clarifying responses. We aimed to create a supportive environment for open discussion, though we cannot be certain everyone felt fully at ease. To mitigate potential limitations, two researchers facilitated the sessions—one managed the process and the other ensured focused discussions and inclusive participation.

Another limitation is that this study exclusively investigated the perspectives of youth healthcare nurses, leaving out perspectives from other key partners involved in the process of prenatal home visits, such as the potential referrers.

## 7 Conclusion

To conclude, there are notable variations in the organisation and execution of prenatal home visits across youth healthcare organisations in the Netherlands. The concept of potential vulnerability remains ambiguous, leading to difficulties in its practical application and an inconsistently defined target population. Besides finding common ground on the definition of potential vulnerability, further research is needed to gain insight into the perspectives of parents and midwives. The quality and effectiveness of prenatal home visits cannot be effectively monitored due to the lack of standardised data registration. Both standardised registration and a clear definition of vulnerability are essential for proper implementation, evaluation, and the continued viability of prenatal home visits.

## Author Contributions

**Roos van Tartwijk:** formal analysis (equal), writing original draft (lead); **Ingrid Staal:** formal analysis (equal), writing original draft (supporting), review and editing (equal); **Yvonne Vanneste:** conceptualisation (lead), methodology (lead), formal analysis (equal), writing original draft (supporting), review and editing (equal).

## Conflicts of Interest

The authors declare no conflicts of interest.

## Data Availability

Data available on request from the authors.

## Appendix A Standards for Reporting Qualitative Research (SRQR)

Reference: *O'Brien BC, Harris IB, Beckman TJ, Reed DA, Cook DA. Standards for reporting qualitative research: a synthesis of recommendations. *Academic Medicine*, Vol. 89, No. 9/Sept 2014. doi: 10.1097/ACM.0000000000000388, https://www.equator‐network.org/reporting‐guidelines/srqr/.

**Table:**

**Title and Abstract**	Page
**Title**—Concise description of the nature and topic of the study Identifying the study as qualitative or indicating the approach (e.g., ethnography, grounded theory) or data collection methods (e.g., interview, focus group) is recommended	1
**Abstract**—Summary of key elements of the study using the abstract format of the intended publication; typically includes background, purpose, methods, results, and conclusions	1
**Introduction**	
**Problem formulation**—Description and significance of the problem/phenomenon studied; review of relevant theory and empirical work; problem statement	2, 3
**Purpose or research questio**n—Purpose of the study and specific objectives or questions	3, 4
**Methods**	
**Qualitative approach and research paradigm**—Qualitative approach (e.g., ethnography, grounded theory, case study, phenomenology, narrative research) and guiding theory if appropriate; identifying the research paradigm (e.g., postpositivist, constructivist/interpretivist) is also recommended; rationale**	4
**Researcher characteristics and reflexivity**—Researchers' characteristics that may influence the research, including personal attributes, qualifications/experience, relationship with participants, assumptions, and/or presuppositions; potential or actual interaction between researchers' characteristics and the research questions, approach, methods, results, and/or transferability	5
**Context**—Setting/site and salient contextual factors; rationale**	4
**Sampling strategy**—How and why research participants, documents, or events were selected; criteria for deciding when no further sampling was necessary (e.g., sampling saturation); rationale**	4, 5
**Ethical issues pertaining to human subjects**—Documentation of approval by an appropriate ethics review board and participant consent, or explanation for lack thereof; other confidentiality and data security issues	5, 6
**Data collection methods**—Types of data collected; details of data collection procedures including (as appropriate) start and stop dates of data collection and analysis, iterative process, triangulation of sources/methods, and modification of procedures in response to evolving study findings; rationale**	4, 5
**Data collection instruments and technologies**—Description of instruments (e.g., interview guides, questionnaires) and devices (e.g., audio recorders) used for data collection; if/how the instrument(s) changed over the course of the study	5
**Units of study**—Number and relevant characteristics of participants, documents, or events included in the study; level of participation (could be reported in results)	5, 6
**Data processing**—Methods for processing data prior to and during analysis, including transcription, data entry, data management and security, verification of data integrity, data coding, and anonymization/de‐identification of excerpts	5
**Data analysis**—Process by which inferences, themes, etc., were identified and developed, including the researchers involved in data analysis; usually references a specific paradigm or approach; rationale**	5
**Techniques to enhance trustworthiness**—Techniques to enhance trustworthiness and credibility of data analysis (e.g., member checking, audit trail, triangulation); rationale**	5
**Results/findings**	
**Synthesis and interpretation**—Main findings (e.g., interpretations, inferences, and themes); might include development of a theory or model, or integration with prior research or theory	6‐14
**Links to empirical data**—Evidence (e.g., quotes, field notes, text excerpts, photographs) to substantiate analytic findings	6‐14
**Discussion**	
**Integration with prior work, implications, transferability, and contribution(s) to the field**—Short summary of main findings; explanation of how findings and conclusions connect to, support, elaborate on, or challenge conclusions of earlier scholarship; discussion of scope of application/generalizability; identification of unique contribution(s) to scholarship in a discipline or field	14‐17
**Limitations**—Trustworthiness and limitations of findings	17
**Other**	
**Conflicts of interest**—Potential sources of influence or perceived influence on study conduct and conclusions; how these were managed	Title page
**Funding**—Sources of funding and other support; role of funders in data collection, interpretation, and reporting	Title page

*The authors created the SRQR by searching the literature to identify guidelines, reporting standards, and critical appraisal criteria for qualitative research; reviewing the reference lists of retrieved sources; and contacting experts to gain feedback. The SRQR aims to improve the transparency of all aspects of qualitative research by providing clear standards for reporting qualitative research.

**The rationale should briefly discuss the justification for choosing that theory, approach, method, or technique rather than other options available, the assumptions and limitations implicit in those choices, and how those choices influence study conclusions and transferability. As appropriate, the rationale for several items might be discussed together.

## Appendix B Good Reporting of a Mixed Methods Study (GRAMMS) Checklist

The GRAMMS Checklist is from O'Cathain A, Murphy E, and Nicholl J. The quality of mixed‐methods studies in health services research. Journal of Health Services Research & Policy. 2008;13(2):92‐8. doi: 10.1258/jhsrp.2007.007074.

**Table:**

Item	Guide questions/description	Page
1	Describe the justification for using a mixed methods approach to the research question	4
2	Describe the design in terms of the purpose, priority and sequence of methods	4
3	Describe each method in terms of sampling, data collection and analysis	4, 5
4	Describe where integration has occurred, how it has occurred and who has participated in it	4, 5, 6
5	Describe any limitation of one method associated with the presence of the other method	4, 5, 6
6	Describe any insights gained from mixing or integrating methods	6

## References

[jan16912-bib-0001] Anraad, C. 2023. Promoting informed decision making about maternal pertussis vaccination. Maastricht University. 10.26481/dis.20230411ca. 37979552

[jan16912-bib-0002] Bekhet, A. K. , and J. A. Zauszniewski . 2012. “Methodological Triangulation: An Approach to Understanding Data.” Nurse Researcher 20, no. 2: 40–43. 10.7748/nr2012.11.20.2.40.c9442. 23316537

[jan16912-bib-0003] Damschroder, L. J. , D. C. Aron , R. E. Keith , S. R. Kirsh , J. A. Alexander , and J. C. Lowery . 2009. “Fostering Implementation of Health Services Research Findings Into Practice: A Consolidated Framework for Advancing Implementation Science.” Implementation Science 4, no. 1: 50. 10.1186/1748-5908-4-50. 19664226 PMC2736161

[jan16912-bib-0004] Dawley, K. , and R. Beam . 2005. “‘My Nurse Taught Me How to Have a Healthy Baby and Be a Good Mother:’ Nurse Home Visiting With Pregnant Women 1888 to 2005.” Nursing Clinics of North America 40: 803–815. 10.1016/j.cnur.2005.08.011. 16324953

[jan16912-bib-0005] De Craen, E. 2024. “Prenatal Home Visits in Youth Health Care: Implementation Research—Final Report.” https://open.overheid.nl/documenten/f7bd4a34‐ee2b‐4907‐ad7d‐2371a7c70edf/file.

[jan16912-bib-0006] Detmar, S. , and M. Wolff de . 2019. The First 1000 Days: Strengthening Early Development; a Literature Review for Municipalities. TNO.

[jan16912-bib-0007] Dutch Government . 2023. “Legal Database. Public Health Act (Wpg). Public Health Act; Chapter II: Public Health Duties, Article 2.i.” https://wetten.overheid.nl/BWBR0024705/2024‐11‐06.

[jan16912-bib-0008] Feijen‐de Jong, E. I. , J. C. Warmelink , M. Dalmaijer , and R. A. van der Stouwe . 2021. “Vulnerability During Pregnancy Is More Than an Imbalance Between Risk Factors and Protective Factors.” TSG 99: 132–136. 10.1007/s12508-021-00308-9.

[jan16912-bib-0009] Greene, J. C. , V. J. Caracelli , and W. F. Graham . 1989. “Toward a Conceptual Framework for Mixed‐Method Evaluation Designs.” Educational Evaluation and Policy Analysis 11, no. 3: 255–274. 10.3102/01623737011003255.

[jan16912-bib-0010] Hope Corbin, J. , J. Jones , and M. M. Barry . 2018. “What Makes Intersectoral Partnerships for Health Promotion Work? A Review of the International Literature.” Health Promotion International 2018, no. 33: 4–26. 10.1093/heapro/daw061. PMC5914378 27506627

[jan16912-bib-0011] Immink, M. M. , K. van Zoonen , N. M. Jager , et al. 2023. “Maternal Vaccination Against Pertussis as Part of the National Immunization Program: A Qualitative Evaluation Among Obstetric Care Providers One Year After the Implementation in December 2019.” BMC Health Services Research 23: 311. 10.1186/s12913-023-09274-1. 36998072 PMC10062680

[jan16912-bib-0012] Loomans, E. M. , A. E. Van Dijk , T. G. M. Vrijkotte , et al. 2013. “Psychosocial Stress During Pregnancy Is Related to Adverse Birth Outcomes: Results From a Large Multi‐Ethnic Community‐Based Birth Cohort.” European Journal of Public Health 23: 485–491. 10.1093/eurpub/cks097. 22850187

[jan16912-bib-0013] Ministry of Health, Welfare and Sport (VWS) . 2018. “Action Program: Promising Start.” Government of the Netherlands. https://www.government.nl/ministries/ministry‐of‐health‐welfare‐and‐sport.

[jan16912-bib-0014] Molloy, C. , R. Beatson , C. Harrop , N. Perini , and S. Goldfeld . 2021. “Systematic Review: Effects of Sustained Nurse Home Visiting Programs for Disadvantaged Mothers and Children.” Journal of Advanced Nursing 77: 147–161. 10.1111/jan.14576. 33038049

[jan16912-bib-0015] O'Brien, B. C. , I. B. Harris , T. J. Beckman , D. A. Reed , and D. A. Cook . 2014. “Standards for Reporting Qualitative Research: A Synthesis of Recommendations.” Academic Medicine 89: 1245–1251. 10.1097/ACM.0000000000000388. 24979285

[jan16912-bib-0016] Popil, I. 2011. “Promotion of Critical Thinking by Using Case Studies as Teaching Method.” Nurse Education Today 31: 204–207. 10.1016/j.nedt.2010.06.002. 20655632

[jan16912-bib-0017] Rutz, S. , I. Abaaziz , M. van der Hulst , and E. Joosse . 2024. A Good Start for Everyone! A Participatory Action Research on Integrated Collaboration Around Prenatal Home Visits in Delft. Hague University of Applied Sciences. https://www.kennisnetwerkjeugdhaaglanden.nl/rapport‐prenataal‐huisbezoek.

[jan16912-bib-0018] Schoonenboom, J. , and R. B. Johnson . 2017. “How to Construct a Mixed Methods Research Design.” Kolner Zeitschrift fur Soziologie und Sozialpsychologie 69: 107–131. 10.1007/s11577-017-0454-1. 28989188 PMC5602001

[jan16912-bib-0019] Soratto, J. , D. E. P. de Pires , and S. Friese . 2020. “Thematic Content Analysis Using ATLAS.Ti Software: Potentialities for Researchs in Health.” Revista Brasileira de Enfermagem 73, no. 3: e20190250. 10.1590/0034-7167-2019-0250. 32321144

[jan16912-bib-0020] The European Parliament and the Council of the European Union . 2016. “Regulation on the Protection of Natural Persons With Regard to the Processing of Personal Data and on the Free Movement of Such Data, and Repealing Directive 95/46/EC (General Data Protection Regulation).”

[jan16912-bib-0021] Then, L. , A. K , J. Ranking , and E. Ali . 2014. “Focus Group Research—What Is It and How Can It be Used.” Canadian Journal of Cardiovascular Nursing 24, no. 1: 16–22. https://pubmed.ncbi.nlm.nih.gov/24660275/. 24660275

[jan16912-bib-0022] Van den Bergh, B. R. H. , M. I. van den Heuvel , M. Lahti , et al. 2020. “Prenatal Developmental Origins of Behavior and Mental Health: The Influence of Maternal Stress in Pregnancy.” Neuroscience and Biobehavioral Reviews 117: 26–64. 10.1016/J.NEUBIOREV.2017.07.003. 28757456

[jan16912-bib-0023] Van den Haak, L. , and E. Struijf . 2021. “Guideline for Prenatal Home Visits by Youth Health Care (JGZ): For Pregnant Women and/or Families in Vulnerable Situations.” NCJ Dutch Centre for Youth Health. https://www.ncj.nl/inspiratie/handreiking‐prenataal‐huisbezoek‐door‐de‐jgz/.

[jan16912-bib-0024] Van Driessche, A. , H. F. van Stel , R. M. Vink , and I. I. E. Staal . 2021. “Assessing Concerns and Care Needs of Expectant Parents: Development and Feasibility of a Structured Interview.” International Journal of Environmental Research and Public Health 18, no. 18: 9585. 10.3390/ijerph18189585. 34574510 PMC8467634

[jan16912-bib-0025] Vanneste, Y. , E. Struijff , and I. I. E. Staal . 2024. “De implementatie van het prenatale huisbezoek door de jeugdgezondheidszorg: een eerste verkenning in de jeugdgezondheidszorg.” 10.61431/zdnz9c15.

[jan16912-bib-0026] Vanneste, Y. T. M. , C. I. Lanting , and S. B. Detmar . 2022. “The Preventive Child and Youth Healthcare Service in The Netherlands: The State of the Art and Challenges Ahead.” International Journal of Environmental Research and Public Health 19: 8736. https://www.mdpi.com/1660‐4601/19/14/8736. 35886585 10.3390/ijerph19148736 PMC9320981

[jan16912-bib-0027] Wulffraat, A. , L. Blanchette , L. Bertens , H. Ernst , L. van der Meer , and H. de Graaf . 2019. “Definition of Vulnerability—Pregnant Women. Action Program: Strong Start Rotterdam.” https://www.dvprijnmond.nl/wp‐content/uploads/2019/10/003‐2019‐09‐02‐Definitie‐Kwetsbaarheid_def.pdf.

[jan16912-bib-0028] Zapart, S. , J. Knight , and L. Kemp . 2016. “'It Was Easier Because I Had Help': Mothers' Reflections on the Long‐Term Impact of Sustained Nurse Home Visiting.” Maternal and Child Health Journal 20, no. 1: 196–204. 10.1007/s10995-015-1819-6. 26497131

[jan16912-bib-0029] Zhe chin, K. , B. Marklund , S. Kylén , and S. Dalemo . 2023. “Extended Prenatal and Postnatal Home Visits in a Vulnerable Area in Sweden—a Pilot Study.” Scandinavian Journal of Primary Health Care 41, no. 4: 486–494. 37910395 10.1080/02813432.2023.2277756 PMC11001331

